# Myxobacteria Are Able to Prey Broadly upon Clinically-Relevant Pathogens, Exhibiting a Prey Range Which Cannot Be Explained by Phylogeny

**DOI:** 10.3389/fmicb.2017.01593

**Published:** 2017-08-22

**Authors:** Paul G. Livingstone, Russell M. Morphew, David E. Whitworth

**Affiliations:** Institute of Biological Environmental and Rural Sciences, Aberystwyth University Aberystwyth, United Kingdom

**Keywords:** microbial predation, isolation, myxobacteria, prey range, pathogen

## Abstract

Myxobacteria are natural predators of microorganisms and the subjects of concerted efforts to identify novel antimicrobial compounds. Myxobacterial predatory activity seems to require more than just the possession of specific antimicrobial metabolites. Thus a holistic approach to studying predation promises novel insights into antimicrobial action. Here, we report the isolation of 113 myxobacteria from samples of soil taken from a range of habitats in mid Wales. Predatory activity of each isolate was quantified against a panel of clinically important prey organisms, including *Klebsiella pneumoniae, Proteus mirabilis, Candida albicans, Enterococcus faecalis*, and three species of *Staphylococcus*. Myxobacterial isolates exhibited a wide range of predation activity profiles against the panel of prey. Efficient predation of all prey by isolates within the collection was observed, with *K. pneumoniae* and *C. albicans* proving particularly susceptible to myxobacterial predation. Notably efficient predators tended to be proficient at predating multiple prey organisms, suggesting they possess gene(s) encoding a broad range killing activity. However, predatory activity was not congruent with phylogeny, suggesting prey range is subject to relatively rapid specialization, potentially involving lateral gene transfer. The broad but patchy prey ranges observed for natural myxobacterial isolates also implies multiple (potentially overlapping) genetic determinants are responsible for dictating predatory activity.

## Introduction

Myxobacteria are social predators, studied extensively for their potential to produce natural products. Their predatory secretions have already been extensively exploited by the pharmaceutical industry, with over 100 core structures and 500 derivatives of novel antibiotics reported in the literature (Weissman and Müller, 2010; Korp et al., 2016). While efficient predation is often assumed to being primarily due to the production of secondary metabolites, there is, however, increasing evidence of the involvement of other mechanisms (Evans et al., 2012; Findlay, 2016; Lloyd and Whitworth, 2017). For example, genome-wide association approaches comparing predatory bacteria with non-predators, have revealed genes unique to predators which seem to have little involvement in secondary metabolite production (Pasternak et al., 2013). Thus, despite the ubiquitous nature of these predators and their therapeutic potential, there remains a dearth of knowledge regarding the mechanisms of predation and prey killing by myxobacteria.

The saprophytic myxobacteria inhabit a variety of soils, which have yielded mesophilic, thermophilic and anaerobic antibiotic-producing organisms (Dawid, 2000; Gerth and Müller, 2005), while marine sampling has also produced myxobacterial predators capable of synthesizing interesting and novel antibiotics (Iizuka et al., 1998). Through the ongoing application of 16S rRNA and whole-genome sequence-based classification methods (for example, Garcia et al., 2010; Sharma et al., 2016a,b), the Myxococcales are currently divided into three sub-orders, 10 families, 30 genera, and around 60 species. Myxobacteria are fastidious organisms, and consequently relatively difficult to work with experimentally. Complex isolation and propagation techniques are required, which impede the isolation and characterization of novel myxobacteria. For instance, most isolates do not grow as smooth suspensions in liquid cultures, which particularly hampers efforts to study their secondary metabolites (Weissman and Müller, 2009), and which in turn promotes a strategy of studying culture extracts and purified products (Charousová et al., 2017).

Among the many aspects of myxobacterial predation, prey range remains particularly poorly understood. Similar isolates can exhibit very different patterns of prey susceptibility (Morgan et al., 2010) and the range of organisms susceptible to the action of purified secondary metabolites can differ from that observed in co-culture experiments (Kunze et al., 2008; Charousová et al., 2017). To date, studies employing co-culture predation assays have tended to either use environmental saprophytes as potential prey, or a small number of human pathogens (Morgan et al., 2010; Evans et al., 2012; Seccareccia et al., 2015).

In this study we took an empirical approach to define the prey range of naturally occurring myxobacteria. We therefore isolated 113 novel myxobacteria, from diverse terrestrial environments in mid Wales, and tested them for antimicrobial activity against a panel of clinically-relevant micro-organisms.

## Materials and Methods

### Soil Sampling and Culture Isolation

Soil samples from various habitats including woodlands, gardens, farmlands, streams, and open fields were collected from the Aberystwyth and Carmarthen areas in West Wales. Approximately, 20–30 g of soil were collected from undisturbed areas avoiding surface soil. Samples were air dried in the laboratory and then inoculated onto culture medium.

Standard isolation methods using WCX and STAN-21 agar (Garcia and Müller, 2014a,b) were employed for bacteriolytic and cellulolytic myxobacteria respectively. Molten WAT agar (0.1% w/v CaCl_2._2H_2_O, 1.5% w/v agar, 20 mM HEPES) at 55°C was supplemented with 2.5% cycloheximide to a final concentration of 25 mg/ml (WCX). WCX plates were spotted with an *Escherichia coli* suspension and allowed to dry before inoculation with soil samples. STAN21 was prepared by mixing two volumes of molten Solution A (0.1% w/v K_2_HPO_4_, 0.002% w/v Yeast Extract, 1.5% w/v agar) to one volume of Solution B (0.1% w/v KNO_3_, 0.1% w/v MgSO_4_.7H_2_O, 0.1% w/v CaCl_2_.2H_2_O, 0.02% w/v FeCl_2_, 0.01% w/v MnSO_4_.7H_2_O) before pouring plates. Small filter paper strips were then placed on the surface of the agar.

Approximately, 1 g of soil was placed in close proximity to the *E. coli* spot on the WCX agar or the filter paper strip on the STAN21 agar. The plates were incubated at 30°C for 2 weeks, examining under a dissection microscope for fruiting bodies and swarming growth every day after the 4th day after incubation. Either fruiting bodies or the agar portion of the advancing edge of the swarm growth was transferred onto fresh water agar and then onto VY-2 agar (0.5% w/v dried baker’s yeast, 0.1% w/v CaCl_2._2H_2_O, 1.5% w/v agar) until pure (Garcia and Müller, 2014a,b). Pure isolates were stored at -80°C.

### 16S rRNA Sequencing and Analysis

Pure cultures were characterized by 16S rRNA sequencing. A ∼1350 bp fragment of the 16S rRNA gene was amplified by PCR using the F27 (AGAGTTTGATCMTGGCTCAG) and R1389 (ACGGGCGGTGTGTACAAG) primers (Hongoh et al., 2003). PCR reactions were carried out with an initial denaturation at 95°C (2 min), and then 35 cycles of denaturation at 95°C (0.5 min), annealing at 55°C (1 min), and extension at 72°C (90 s), with a final extension at 72°C (10 min). PCR products were visualized by agarose gel electrophoresis and purified using an EZ-10 spin column PCR purification kit (Bio Basic). Purified PCR products were then sequenced from both ends, and then assembled for complete coverage using BioEdit (Hall, 1999). Assembled 16S rRNA sequences were submitted as queries against the EzTaxon database of 16S sequences to identify the classified organisms with the most similar 16S genes. 16S sequences were aligned using MEGA7 (Kumar et al., 2016) and phylogenetic trees constructed using the Kimura-2 parameter model, with 500 bootstraps. The 16S rRNA gene sequences from myxobacterial type strains were included for benchmarking.

### Predation Assays

A lawn culture method was employed in this assay (Morgan et al., 2010). Briefly, 10 prey organisms (**Table 1**) were grown in Luria Bertani (LB) broth for 16–18 h and subjected to centrifugation at 4000 *g* for 30 min. Sedimented cells were then washed and resuspended into TM buffer (50 mM Tris, pH 7.8, 10 mM MgSO_4_). A 1 ml volume of the washed cells was poured and spread onto a 14 cm diameter WAT agar plate and dried to form a uniform lawn. Myxobacterial isolates were grown in AMB broth (Garcia and Müller, 2014a,b) at 30°C for 5–7 days to obtain a dense culture (OD_600_ of ∼2). Cultures were then subjected to centrifugation at 4000 × *g* for 30 min, the pellet was washed in TM buffer and 10 μl of the cell pellet spotted onto the prey lawn. Plates were incubated and the diameter of the zone of swarming was recorded on day 4 as a measure of predatory activity. Predatory activity data for the 10 prey organisms were clustered using the hierarchal clustering method in R (Everitt, 1974).

**Table 1:** Prey organisms used in the study.

Organism	Gram stain	Order	Strain/origin
*Escherichia coli*	Negative	Enterobacteriales	ATCC 25922
*Klebsiella pneumoniae*	Negative	Enterobacteriales	ATCC 700603
*Proteus mirabilis*	Negative	Enterobacteriales	NCTC 10975
*Pseudomonas aeruginosa*	Negative	Pseudomonadales	ATCC 27853
*Staphylococcus aureus*	Positive	Bacillales	ATCC 29213
*Staphylococcus epidermidis*	Positive	Bacillales	NCTC 11047
*Staphylococcus saprophyticus*	Positive	Bacillales	Wild-type laboratory strain
*Enterococcus faecalis*	Positive	Lactobacillales	ATCC 29212
*Bacillus subtilis*	Positive	Bacillales	ATCC 6633
*Candida albicans*	Positive	Saccharomycetales	NCTC 32

## Results

### A Collection of Novel Welsh Myxobacterial Isolates

In total, 113 strains with unique phenotypes and/or 16S rRNA gene sequences were isolated from 77 soil samples from the Carmarthen and Aberystwyth areas of the United Kingdom (**Supplementary File 1**). When samples gave more than one isolate, isolates were required to be morphologically distinct, to ensure the collection was non-redundant. As observed in other studies (Zhang et al., 2013; Charousová et al., 2017), isolates were predominantly *Corallococcus* spp. (70%), and *Myxococcus* spp. (24%) while *E. coli* baiting was found to be the most efficient method for myxobacterial isolation. There was no obvious relationship between the environment/location sampled and the species of myxobacteria isolated.

The fruiting bodies of *Myxococcus* spp. isolates were generally large, spherical, yellow to orange in color, and slimy in appearance, whilst the vegetative cells were slender with tapering ends, producing a thin film of yellow swarming growth on VY-2 medium. *Corallococcus* spp. isolates produced smaller fruiting bodies in groups, had vegetative cells which were long with tapering ends and produced colonies which appeared as thin films of colorless to brown swarming growth. *Pyxidicoccus* spp. isolates produced smaller fruiting bodies and colonies, while *Sorangium* spp. isolates grew as orange colonies, degrading the cellulose when growing on filter paper and burrowing into the media on agar plates. *Sorangium* spp. isolates formed orange fruiting bodies, and their vegetative cells were short and blunt-ended.

### Isolates belong to One of Six Discrete Phylogenetic Clusters

**FIGURE 1:**
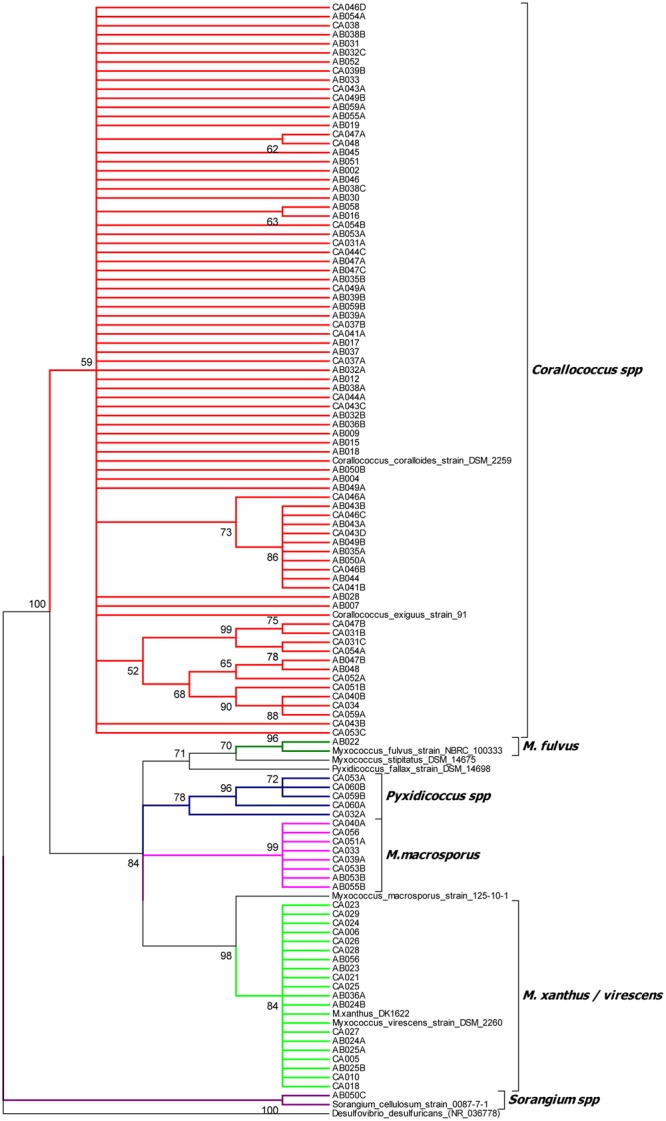
16S rRNA gene sequence tree of 113 myxobacterial isolates and selected myxobacterial type strains. The tree was rooted against the non-myxobacterium Deltaproteobacterium *Desulfovibrio desulfuricans* and clades with less than 50% boostrap support were collapsed.

The isolates’ 16S rRNA gene sequences were used to identify the closest taxon for each isolate (**Supplementary File 1**) and to construct a phylogenetic tree, which also included the 16S rRNA sequences of formally classified myxobacteria (**Figure 1** and **Supplementary File 2**). The phylogenetic tree placed the 113 isolates into 6 clusters with strong bootstrap support, which agreed in every case with the taxon assignments generated by EzTaxon.

Cluster 1 (*Corallococcus* spp.) included 79 isolates all with EzTaxon assignments of *C. exiguus.* The cluster also included *C. coralloides*. The *C. coralloides* and *C. exiguus* type strains have a 16S rRNA sequence similarity of 99.9%, leading to claims that the two species are the same albeit with some trivial differences in morphological features (Garcia et al., 2010). Within cluster 1 four sub-clusters with strong bootstrap support can be seen, containing between 2 and 11 isolates. No type strains localized within these sub-clusters, precluding a more specific assignment. A separate tree of the Cluster 1 isolates is available in **Supplementary File 3**.

Cluster 2 (*M. xanthus/virescens*) contains 19 isolates of which EzTaxon assigned 17 as *Myxococcus virescens* and two as *Myxococcus xanthus*. In the phylogenetic tree they formed a single clade, which also included the *M. xanthus* and *M. virescens* type strains. A separate tree of Cluster 2 isolates is also available in **Supplementary File 3**.

Cluster 3 (*M. macrosporus*) and Cluster 4 (*Pyxidicoccus* spp.) included eight and five isolates respectively. Seven of the eight isolates in the *M. macrosporus* cluster were identified as *C. macrosporus* by EzTaxon (reassigned as *M. macrosporus* by Garcia et al., 2010), while all six members of the *Pyxidicoccus spp*. cluster were identified as *P. fallax*. Neither the *P. fallax* nor *M. macrosporus* type strains grouped within their eponymous clusters, on average sharing 16S gene sequence similarities of <99% with cluster members.

Clusters 5 and 6 each contained a single isolate. Cluster 5 (*M. fulvus*) formed a clade including the *M. fulvus* type strain (95% bootstrap support), close to *M. stipitatus* but distant from the *M. xanthus/virescens* cluster. The Cluster 6 (*Sorangium* spp.) isolate grouped with *Sorangium cellulosum*, albeit with 16S sequence similarity of <98%.

### Isolates Exhibit a Broad Range of Predatory Activities against Pathogenic Microbes

**FIGURE 2:**
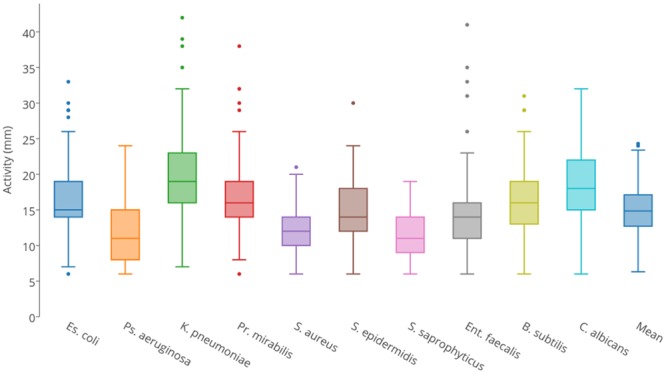
Box and whisker plots of isolates’ predatory activity (zone of killing diameter in mm) illustrating the variation in predatory activity exhibited by all isolates for each of the 10 prey organisms.

Each isolate was tested for predatory activity against a panel of 10 clinically important ‘prey’ organisms, which included Gram-negative bacteria, Gram-positive bacteria, and yeast. Isolates were inoculated onto lawns of prey and predation activity was defined as the diameter of the resulting zones of predation after 4 days (**Supplementary File 1**). **Figure 2** summarizes the observed predatory activity of the 113 isolates from the perspective of each prey. Prey pathogens exhibited differing susceptibility to the predators, for instance *Pseudomonas aeruginosa* was on average more recalcitrant to predation by the panel of myxobacteria than *Klebsiella pneumoniae*.

Predatory activity varied for each prey organism used. With all prey, the minimum activity of any isolate (zone of predation) was 6 mm in diameter, which was the size of the initial inoculum, thus indicating no predatory activity. Mean activities were between 11 and 20 mm, with maximum observed activities varying between 19 mm (*Staphylococcus saprophyticus*) and 42 mm (*K. pneumoniae*). Predatory activity also varied substantially between isolates. Three isolates (all members of the *Corallococcus* spp. cluster) were particularly poor predators, with mean activities against the 10 prey of <10 mm. The other isolates were defined as ‘moderate predators,’ exhibiting a continuum of mean predatory activity, ranging from 10.0 to 20.6 mm. However, four isolates (two from each of the *Corallococcus* spp. and *M. xanthus/virescens* clusters) were particularly good predators, with mean activities between 23.2 and 24.3 mm. Surprisingly, the model myxobacterium *M. xanthus* DK1622 is a relatively poor predator compared to the newly isolated organisms, with a mean activity of just 11.5 mm, despite the *M. xanthus/virescens* isolates being the best cluster of predators, with a mean activity of 16.81 mm.

The predators with the greatest activities against individual prey tended to be efficient at killing multiple prey, including Gram-negative and -positive organisms. However, they often exhibited overlapping prey ranges. For instance CA010 was the single best predator of *E. coli, K. pneumoniae, Proteus mirabilis* and *Enterococcus faecalis*, and CA054B was the top predator against *P. aeruginosa, S. saphrophyticus, Bacillus subtilis* and *Candida albicans*, and both strains were amongst the top five best predators of *Staphylococcus aureus*. Similarly, CA029 was amongst the top five best predators for five prey, including Gram-negative, Gram-positive and yeast strains, but was a poor predator of *B. subtilis*. Occasionally, only moderate predators exhibited potent activity against individual prey species, for instance AB050C has a very typical mean activity of 15.3 mm, yet it is amongst the top five predators of *S. saprophyticus*.

### Predatory Activity and Prey Susceptibility Are Only Partially Dictated by Phylogeny

To investigate relationships between the predatory activity profiles of different isolates, the predation activity matrix was clustered, resulting in a tree where the closest leaves belonged to isolates with the greatest similarity in predatory activity against the 10 prey organisms – a ‘predation’ tree. The data were also clustered in the orthogonal direction, resulting in a tree of prey organisms, where the closest leaves were those prey which showed the most similar pattern of susceptibility to predation by the 113 isolates – a ‘susceptibility’ tree.

**FIGURE 3:**
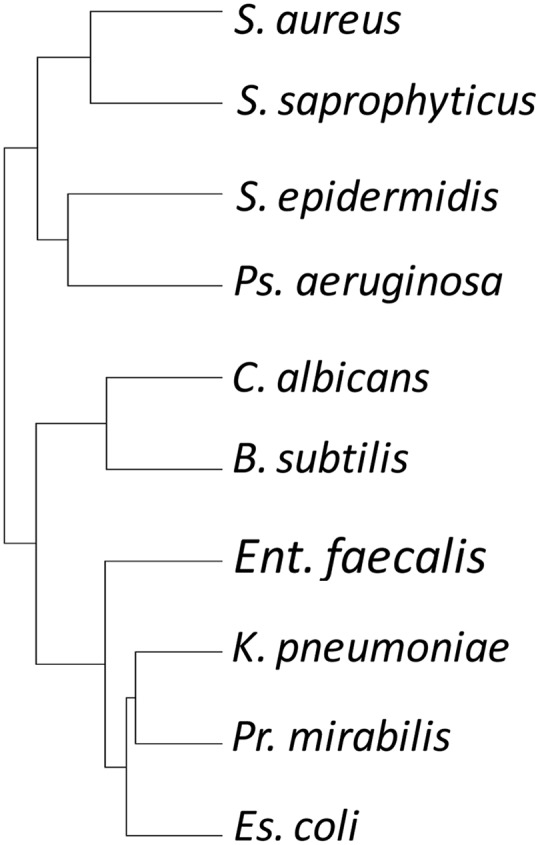
Hierarchical clustering tree of prey organisms’ susceptibility profile to attack by myxobacterial isolates. Susceptibility to attack does not recapitulate the phylogeny of the prey organisms.

The susceptibility tree (**Figure 3**) largely reflected phylogeny, with for instance Gram-negative *E. coli, P. Mirabilis*, and *K. pneumoniae* grouping together. However, that clade also grouped with *E. faecalis*, a Gram-positive Firmicute. Another Firmicute, *B. subtilis*, grouped with the fungus *C. albicans*, while the three *Staphylococcus* strains grouped together, but with *P. aeruginosa* (**Figure 3**). Thus susceptibility to predation is only partially due to phylogeny, implying that susceptibility determinants can either be transferred laterally between organisms, or are multifactorial.

**FIGURE 4:**
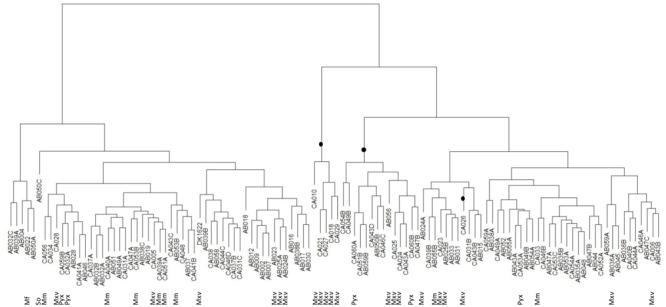
Hierarchical clustering tree of myxobacteria isolate predation profiles. Clades highlighted with a black dot represent the 20 ‘best’ predators. So = *Sorangium* spp., Mf = *M. fulvus*, Mxv = *M. xanthus/virescens*, Mm = *M. macrosporus*, Pyx = *Pyxidicoccus* spp., and unlabeled leaves were all *Corallococcus* spp.

Similarly, the predation tree (**Figure 4**) shows that the predatory profile is not merely a consequence of phylogeny. As expected, better predators tended to group together, as did the poorer predators. Of the 20 ‘best’ predators with the highest mean predation activities, 19 grouped together in the predation tree in two distinct clades. One of the clades contained five *M. xanthus/virescens* isolates, however, the other clade contained two *Pyxidicoccus* spp., three *M. xanthus/virescens* and nine *Corallococcus* spp. isolates (**Figure 4**). Most other clades were dominated by *Corallococcus* spp. isolates. However, some of those clades nevertheless had sub-groups containing isolates belonging to the *Pyxidicoccus* spp., *M. macrosporus*, and *M. xanthus/virescens* clusters (**Figure 4**). Thus, it appears that predatory range is not strongly influenced by phylogeny, implying evolution of these organisms involves relatively frequent acquisition/loss of the predation factors which determine prey range.

## Discussion

Myxobacteria are natural predators of diverse microorganisms, and are consequently the subject of concerted efforts to identify novel antimicrobial compounds for clinical applications (Korp et al., 2016). However, there is increasing evidence that effective predation is more than just the consequence of possessing particular secondary metabolites (Xiao et al., 2011; Findlay, 2016; Muñoz-Dorado et al., 2016). This study adopted a more holistic approach to antimicrobial discovery by investigating the predatory activity of novel myxobacterial isolates toward 10 diverse prey organisms, which included nine clinically-important pathogens and the model Gram-negative and Gram-positive organisms *E. coli* and *B. subtilis*. Myxobacterial co-operative behaviors have been studied for several decades, particularly motility and multicellular development (Whitworth, 2008; Muñoz-Dorado et al., 2016). However, only a few studies have systematically investigated predation and prey range, having instead focused on prey organisms that myxobacteria may encounter in their natural environment (Morgan et al., 2010).

In the current study 113 myxobacterial strains were isolated, the majority of which were *Corallococcus* spp. and *M. xanthus/virescens*; an isolation bias seen in other studies (Zhang et al., 2013; Mohr et al., 2016; Charousová et al., 2017). We were unable to isolate members of the Nannocystineae sub-order, with all our isolates belonging to the Cystobacterineae, except for one *Sorangium* spp. isolate belonging to the Sorangineae sub-order. Although diverse myxobacteria have been isolated from a wide range of habitats (including psychrophiles from Antarctic soil and acidophilic myxobacteria from peat bogs), we concentrated our sampling on temperate cultivated topsoil, a habitat known to be particularly rich in predatory myxobacteria (Dawid, 2000).

The phylogenetic relationships of myxobacteria are still not clear at the genus, species and family levels, with several examples of formally assigned names being at odds with 16S phylogenies and other taxonomic markers, necessitating study-specific ‘functional’ phylogenies or reclassification (Garcia et al., 2010; Whitworth, 2015; Sharma et al., 2016b; Awal et al., 2017). While 16S rRNA sequencing is generally a robust method for bacterial taxonomy, it is of limited use for classifying closely related strains within the same genus, which benefit from further analysis by complementary methods such as multi-locus sequence typing (MLST) or fatty acid methyl ester (FAME) analysis (Zhang et al., 2013). To avoid conflicting with published phylogenies, we instead binned our isolates into six groups on the basis of 16S sequence-based taxon assignment (EzTaxon) and clustering on phylogenetic trees, which gave consistent assignments for all isolates.

Within our *Corallococcus* spp. cluster of isolates, there was a high degree of diversity, with isolates having as little as 97.3% sequence similarity to *Corallococcus* type strains and several sub-clusters with strong bootstrap support and no type strain members. High genetic diversity within the *Corallococcus* spp. has also been noted by other studies using housekeeping genes (Stackebrandt and Päuker, 2005; Stackebrandt et al., 2007) suggesting the genus may actually be an agglomeration of multiple genera. Our phylogeny also supports the proposed reassignment of *M. fulvus* and *M. stipitatus* as *Pyxidicocci* (Garcia et al., 2010).

For each of the 10 prey organisms tested, isolates present in the collection were able to predate upon every organism with an activity of 19 mm or more. Mean activity was relatively low against *S. aureus, S. saprophyticus*, and *P. aeruginosa* (albeit with a mean activity of 11.7 mm), and was generally highest against *C. albicans* and *K. pneumoniae* (mean activity of 19.0 mm). With increasing frequencies of antimicrobial resistance (Bowers and Huang, 2016; Poirel et al., 2017), identification of novel isolates which are able to efficiently predate these pathogens offers the hope of harnessing their predatory activity for use in the clinic.

It is impossible to speculate about the mechanisms employed in the predation of different prey by the various isolates described here. However, mechanistic studies have illuminated some features of predation by particular myxobacteria and/or identified metabolites with antimicrobial activity. *M. xanthus* has been shown to kill *E. coli* through the secretion of both myxovirescin (which inhibits type II signal peptidase) and outer membrane vesicles (Xiao et al., 2012; Berleman et al., 2014). Outer membrane vesicles are packed with hydrolytic enzymes and secondary metabolites, and secretion of such a cocktail of predatory factors may explain the broad prey range exhibited by most myxobacteria, potentially reducing the likelihood of resistance developing (Whitworth, 2011; Berleman et al., 2014; Whitworth et al., 2015).

*Bacillus subtilis* responds to attack by *M. xanthus* with the secretion of bacillaene and by sporulating within predation-resistant megastructures (Müller et al., 2014, 2015). *Corallococcus* spp. are known to produce diverse secondary metabolites, including the corallopyronins, corallozines, and coralmycins (Schmitz et al., 2014; Schäberle et al., 2015; Kim et al., 2016). Coralmycins exhibit some antibiotic activity against Gram-negative bacteria, but are particularly active against Gram-positive bacteria (Kim et al., 2016). *Pyxidicoccus* spp. produce macrolides which are able to kill *S. aureus* (Schieferdecker et al., 2014; Surup et al., 2014), while *Sorangium cellulosum* produces sorangicins, ripostatins, etnangiens and thuggacins, which are particularly active against Gram-positive bacteria and yeast (Weissman and Müller, 2009, 2010; Schäberle et al., 2014).

There appears to be a subtle but potentially important dichotomy in the myxobacterial antimicrobial literature. *Myxococcus* spp. are described as efficient predators of Gram-negative, but with only variable activity against Gram-positive bacteria (Gerth and Müller, 2005; Morgan et al., 2010; Xiao et al., 2011; Müller et al., 2014). Conversely, crude extracts of secondary metabolites from these organisms typically show greater activity against Gram-positive than against Gram-negative bacteria (Morgan et al., 2010; Charousová et al., 2017). It would be interesting to test whether the antimicrobial activities manifested by the isolates described here are also exhibited by cell extracts made from those isolates. Is predatory prey range entirely a consequence of the production of particular secondary metabolites, or (as seems more likely) is predatory activity the result of complex processes involving not only secondary metabolites, but many other factors?

Phylogeny is not a good predictor of predatory activity, from the perspective of both predator and prey (**Figures 3, 4**). This could be a consequence of several (not mutually exclusive) scenarios. The acquisition of predatory genes from other myxobacteria, for instance by horizontal transfer, would tend toward uncoupling predatory phenotype from phylogeny. Rapid evolution to individual micro-niches and their resident microbial prey fauna (a considerable selective pressure for predatory organisms) would result in accelerated evolution of predation/susceptibility genes (convergently and/or divergently potentially), also reducing the consequences of the ancestral lineage. If predatory activity was a consequence of multiple synergistic processes, then independent segregation of the genes involved would lead to a particularly patchy mosaic of predatory activity and prey susceptibility. Horizontal transfer is known to have molded the genomes of contemporary myxobacteria (Goldman et al., 2006, 2007; Whitworth, 2015), and, while some secondary metabolites are unique to certain taxonomic groups of Myxobacteria, there are some which are found in disparate genera (Weissman and Müller, 2009, 2010; Schäberle et al., 2014; Korp et al., 2016).

If we wish to understand the mechanisms of predation and prey range for exploitation in the clinic, it is clearly necessary to look beyond the predatory mechanisms employed by individual type strains. Genome-wide association studies could be employed to identify candidate genes whose presence correlates with predatory activity, and to that end we are currently engaged in sequencing the genomes of the isolates described here.

## Author Contributions

DW and RM conceived the project and supervised its completion, PL designed and performed the experiments, DW and PL interpreted the data, PL drafted the manuscript, DW, RM, and PL edited the manuscript.

## Conflict of Interest Statement

The authors declare that the research was conducted in the absence of any commercial or financial relationships that could be construed as a potential conflict of interest.

## Supplementary Material

FILE S1 Isolate classification and predation activity data.

FILE S2 16S rRNA gene sequence distance tree of all isolates and selected type strains. The scale shows the per base substitution rate.

FILE S3 16S rRNA gene sequence trees of Cluster 1 (*Corallococcus* spp.) and Cluster 2 (*Myxococcus* spp.) isolates. 16S rRNA gene sequences of the isolates are available through www.ncbi.nlm.nih.gov under accession references MF163277-MF163389.
