# Supplementary figures and images for: Myxobacteria Are Able to Prey Broadly upon Clinically-Relevant Pathogens, Exhibiting a Prey Range Which Cannot Be Explained by Phylogeny
Source: Front Microbiol. 2017 Aug 22;8:1593. doi: 10.3389/fmicb.2017.01593 (PMC5572228; doi:10.3389/fmicb.2017.01593)

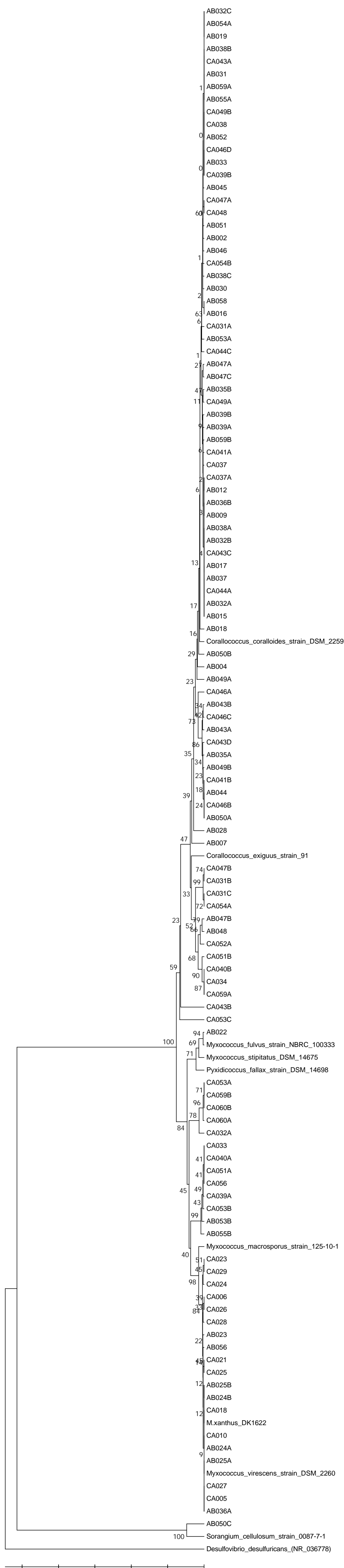

Supplement: FILE S2 — 16S rRNA gene sequence distance tree of all isolates and selected type strains. The scale shows the per base substitution rate. [file Data_Sheet_2.PDF]
