# Supplementary material for: Myxobacteria Are Able to Prey Broadly upon Clinically-Relevant Pathogens, Exhibiting a Prey Range Which Cannot Be Explained by Phylogeny
Source: Front Microbiol. 2017 Aug 22;8:1593. doi: 10.3389/fmicb.2017.01593 (PMC5572228; doi:10.3389/fmicb.2017.01593)
Supplement: FILE S3 — 16S rRNA gene sequence trees of Cluster 1 (Corallococcus spp.) and Cluster 2 (Myxococcus spp.) isolates. 16S rRNA gene sequences of the isolates are available through www.ncbi.nlm.nih.gov under accession references MF163277-MF163389. [file Data_Sheet_3.DOCX]

*Corallococcus spp. – Cluster 1 Tree*

*Myxococcus spp. – Cluster 2 Tree*

**
